# Spatial-Frequency Decoupling Alignment Encoding for Remote Sensing Change Detection

**DOI:** 10.3390/s26061979

**Published:** 2026-03-21

**Authors:** Xu Zhang, Yue Du, Weiran Zhou, Kaihua Zhang

**Affiliations:** 1 School of Electronics and Information Engineering, Suzhou Polytechnic University, Suzhou 215104, China; 2 Information Center, Suzhou Polytechnic University, Suzhou 215104, China; 91822@jssvc.edu.cn; 3 School of Computer Science, Nanjing University of Information Science and Technology, Nanjing 210044, China; zweiran73@gmail.com (W.Z.);

**Keywords:** remote sensing change detection, multi-scale high-frequency interaction, dual-domain alignment fusion, position-aware low-frequency enhancement, selective fusion attention

## Abstract

Existing remote sensing change detection methods often struggle to accurately capture the contours of complex change targets and subtle textural differences. This makes it difficult to effectively distinguish between the boundaries of change targets and the background. To address this challenge, we propose a novel method called spatial-frequency decoupling alignment encoding (SDA-Encoding), which is designed to fully leverage information from both the spatial and frequency domains. Specifically, we first use a Transformer encoder to extract bi-temporal features. Next, we apply wavelet transform to decouple these features into low-frequency and high-frequency components. In the multi-scale high-frequency interaction (MHI) module, we combine local spatial enhancement using spatial pyramid pooling with cross-scale dependency supplementation via the dual-domain alignment fusion (DAF) module. Meanwhile, in the position-aware low-frequency enhancement (PLE) module, spatial position sensitivity is restored using coordinate attention, and region-level contextual dependencies are captured through the selective fusion attention (SFA) module. Finally, the two frequency-domain branches are complementarily fused within the spatial domain to achieve unified detection of both fine-grained and structural changes. Experimental results on three benchmark datasets demonstrate the significant performance improvements of SDA-Encoding.

## 1. Introduction

Remote sensing (RS) image data has experienced tremendous growth in recent years. Currently, change detection (CD) technologies are being widely applied across various domains, including urbanization monitoring [1,2,3], disaster assessment [4,5,6], and agricultural monitoring [7,8,9].

In recent years, CD methods have evolved from traditional approaches [10,11,12,13,14,15,16,17,18] to deep learning-based techniques. Traditional methods rely on hand-crafted features and lack end-to-end optimization, which limits their robustness when dealing with complex noise and diverse real-world scenarios. Nowadays, deep learning methods have increasingly become a focal point of research in remote sensing image change detection [19,20,21,22,23,24,25,26]. Deep models, such as deep belief networks (DBNs) [27], stacked autoencoders (SAEs) [28], and convolutional neural networks (CNNs) [29], have demonstrated superior capabilities in extracting abstract and discriminative features for RSCD. While CNNs effectively preserve spatial information through local connectivity, their limited receptive fields constrain their ability to model global context and handle complex scenes. To overcome this limitation, recent research has introduced methods like dilated convolutions [30,31], spatial pyramid pooling [32,33], and attention mechanisms [34,35] for global context modeling. However, these methods still aggregate information locally. Transformer-based models, by leveraging self-attention, capture long-range dependencies and holistic scene context, resulting in significant improvements for RSCD. Recent advancements, including the Vision Transformer (ViT) [36], Swin Transformer [37], and hybrid CNN-Transformer architectures [38,39], further enhance both efficiency and detection accuracy.

**Figure 1:**
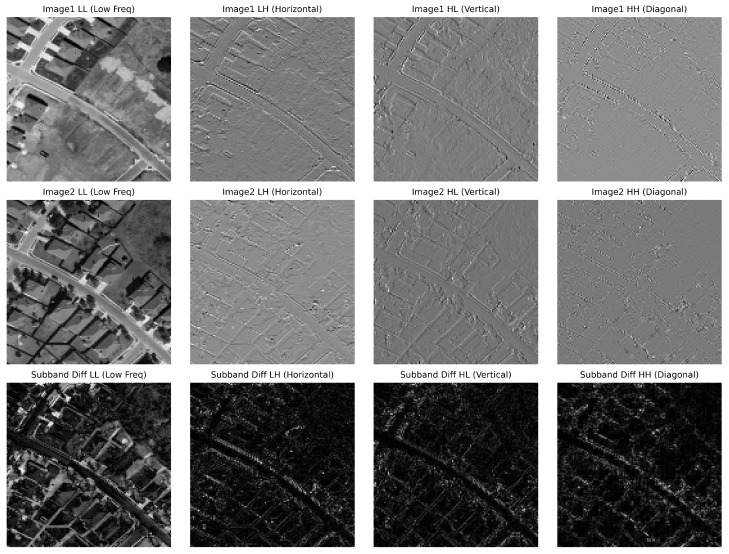
Understanding images in the frequency domain.

In RSCD, the current networks effectively integrate both global and local spatial information. However, they typically rely solely on spatial-domain segmentation without explicitly incorporating frequency-domain features. Illumination variations and shadows in RS images are often misinterpreted as genuine land cover changes in the spatial domain, while blurred regions, lacking stable texture features, are frequently misclassified as unchanged. In signal processing theory, decoupling an image into different frequency components provides a clearer understanding of its content. As shown in Figure 1, the first two rows illustrate the effects of decomposing the image into low-frequency and high-frequency components, with key features gradually becoming more apparent. The low-frequency component reveals the overall structure and layout of the image, while the high-frequency component highlights edges and finer details. The final row presents the differences between the two images across various wavelet subbands, demonstrating that frequency-domain information is more effective in addressing issues such as lighting changes and shadows. Additionally, it plays a crucial role in extracting the image’s structure and boundaries more effectively. Moreover, frequency-domain features are more sensitive to changes in complex regions [40,41,42]. Therefore, actively incorporating frequency-domain information into spatial segmentation networks could improve the model’s robustness, particularly in scenarios involving foreground–background blurring.

Inspired by the insights above, we propose spatial-frequency decoupling alignment encoding (SDA-Encoding). Specifically, in the MiT-based bi-temporal fusion stage, spatial-domain features are first extracted using a Transformer encoder. Through early fusion, a bi-temporal feature space with cross-scale consistency is then constructed, forming the foundation for spatial-frequency decoupling. In the multi-scale high-frequency interaction (MHI) module, recognizing that high-frequency features are sensitive to local geometric structures, we incorporate a spatial pyramid pooling (SPP) [43] module, which introduces multi-scale convolutional kernels to enhance sensitivity to texture variations at different scales. Additionally, high-frequency features often exhibit cross-regional spatial dependencies that convolution alone cannot effectively capture. To address this, we design a dual-domain alignment fusion (DAF) module to perform both spatial and cross-scale enhancements on high-frequency features, enabling the simultaneous capture of local texture variations and long-range edge dependencies. In the position-aware low-frequency enhancement (PLE) module, to enhance positional awareness and multi-scale structural representation for capturing region-level changes, we incorporate a coordinate attention (CA) [44] module. This explicitly encodes spatial position and directional information within the low-frequency features. Additionally, the selective fusion attention (SFA) module is used to model long-range dependencies by jointly learning spatial information in the spatial domain and low-frequency structural features. This strengthens the global structural awareness of low-frequency features, enhancing their ability to capture large-scale changes. Finally, a segmentation head is employed to aggregate the features and generate the final CD results for bi-temporal images.

The contributions of our work can be summarized as follows:

1. An MHI module is designed to enhance high-frequency components by applying spatial and multi-scale enhancements, thereby strengthening fine-grained texture variations. This process transforms these variations into more structured and distinguishable feature cues, improving the model’s ability to detect subtle changes.
2. A PLE module is designed to explicitly restore spatial positional structures, enhancing the structural integrity and consistency of low-frequency features. This enables the model to generate low-frequency change representations that are more sensitive to large-scale changes, thereby improving its ability to discriminate extensive area changes.
3. We introduce SDA-Encoding, a framework that extracts multi-scale features and effectively harnesses information from both the spatial and frequency domains. The framework then aligns these features using multi-scale axial convolutions and dual cross-attention, ultimately generating a CD map.

## 2. Related Works

### 2.1. CNN-Based CD Methods

CNNs have become the dominant approach in RSCD, significantly outperforming traditional methods that rely on hand-crafted features. Among these, encoder–decoder architectures are especially popular due to their effectiveness in generating pixel-wise change maps. For instance, FCN [45] pioneered the use of fully convolutional networks (FCNs) for optical image CD. UNet++ [46] improves edge accuracy by incorporating nested skip connections. Beyond end-to-end approaches, early works like FC-Siam-diff [47] generated different images using Siamese networks but struggled with spectral noise. Recent advancements leverage attention mechanisms to enhance the reliability of the generated images. For example, DMINet [48] integrates self-attention and cross-attention mechanisms to effectively capture spatiotemporal relationships across different feature levels. SEIFNet [49] incorporates CA and CBAM [50] modules to capture global contextual features while effectively emphasizing changed objects. SFCF-Net [51] introduces a novel approach for change detection in high-resolution drone images, effectively addressing noise interference and semantic inconsistency by incorporating multiple specialized modules.

### 2.2. Transformer-Based Methods

The remarkable success of transformer models in computer vision tasks has garnered significant attention from the RSCD community, sparking investigations into their potential applications in CD tasks. For instance, ChangFormer [52] leverages pure Transformer blocks to capture pixel-wise long-range dependencies between bi-temporal images through temporal self-attention, effectively overcoming the limitations of local receptive fields inherent in CNNs. STADE-CDNet [53] combines Transformer and LSTM architectures, with the Transformer encoder extracting global features and the LSTM specifically learning inter-phase state transitions, resulting in computationally efficient representations. BIT [38] is a hybrid architecture and utilizes ResNet18 [54] to capture local details, while the Transformer’s cross-attention mechanism is employed to align global context effectively. It is a dual-branch transformer framework [55] that tackles the challenges of ground and remote sensing image matching by incorporating panoramic sky perception, geometric projection transformation, and gradient-aware weighting modules. This approach significantly enhances geo-location accuracy across varying viewpoints.

### 2.3. Frequency-Based Remote Sensing Image Processing

In recent years, frequency-domain-based methods for remote sensing image processing have gained considerable attention, demonstrating significant advantages in tasks such as image fusion, scene classification, and dehazing [56,57,58,59].

In the field of CD, frequency-domain-related methods have also emerged continuously. FTAN [60] improves the model’s sensitivity to change regions by fusing multi-scale spatial and frequency features. FDTNet [61] enhances the ability to extract both global and local information in complex scenes by integrating frequency-domain processing capabilities with a Transformer architecture. CDC2F [62] achieves continuous-scale change identification through cross-domain structures. Additionally, DDLNet [63] has achieved good results in change detection tasks by combining frequency-domain enhancement modules with spatial-domain recovery modules. RACDNet [64] addresses the resolution disparity issue and improves the change detection accuracy of high-resolution remote sensing images by introducing a gradient-enhanced amplification module and a dual-domain discrepancy learning module.

Compared to existing change detection methods that rely solely on spatial domains or simple frequency fusion, SDA-Encoding introduces a decoupled and aligned frequency enhancement strategy. While methods like FTAN and FDTNet incorporate frequency features, they do not specifically optimize the distinct roles of high- and low-frequency components. SDA-Encoding addresses this gap through two key designs. The multi-scale high-frequency interaction (MHI) module enhances sensitivity to local textures via multi-scale convolutions, while also capturing long-range edge dependencies through dual-domain alignment fusion (DAF). This overcomes the limitations of traditional convolutions in modeling global edge information. Meanwhile, the position-aware low-frequency enhancement (PLE) module encodes spatial position using coordinate attention and reinforces global structural awareness via selective fusion attention (SFA). This effectively mitigates interference from illumination changes and shadows in low-frequency representations. By shifting frequency features from “passive fusion” to “active decoupling and alignment,” SDA-Encoding captures subtle texture changes while robustly identifying large-scale regions, offering enhanced robustness and discriminability in CD tasks.

## 3. Methods

### 3.1. Overview

**Figure 2:**
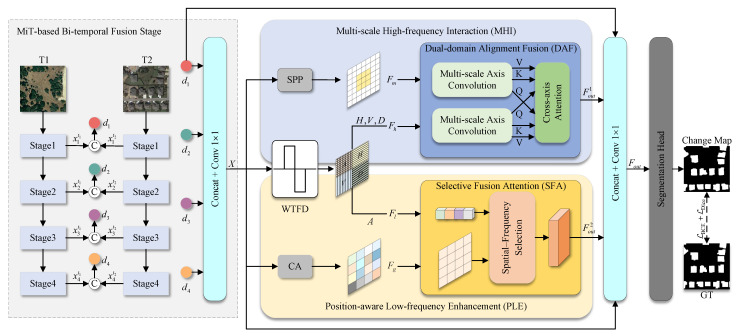
Architecture of the proposed SDA-Encoding.

In this paper, we propose spatial-frequency decoupling alignment encoding (SDA-Encoding) for RSCD. As shown in Figure 2, we adopted the Mix Transformer (MiT) [65] to extract bi-temporal features. At each stage, the feature maps are downsampled to half the resolution of the previous stage. Given temporally paired images $\left\{T_{1},T_{2}\in R^{3\times H\times W}\right\}$, the output bi-temporal features at the four stages can be represented as $\{x_{i}^{t_{1}},x_{i}^{t_{2}}\in R^{C_{i}\times \frac{H}{2^{i+1}}\times \frac{W}{2^{i+1}}}{\}}_{i=1}^{4}$. Subsequently, we concatenate the features and apply convolutional operations to generate the temporal difference features $\{d_{i}\in R^{2C_{i}\times \frac{H}{2^{i+1}}\times \frac{W}{2^{i+1}}}{\}}_{i=1}^{4}$. This process can be formulated as follows:

(1) $$d_{i}=\mathrm{cat}\left(x_{i}^{t_{1}},x_{i}^{t_{2}}\right),$$

where cat(·) represents the feature concatenation operation.

The feature extractor in the first stage generates four multi-scale features, denoted as $\{d_{i}\in R^{2C_{i}\times \frac{H}{2^{i+1}}\times \frac{W}{2^{i+1}}}{\}}_{i=1}^{4}$. Then, $d_{i=2}^{4}$ are resized to the same size as $d_{1}$, and all features are subsequently merged. This procedure can be formulated as:

(2) $$X=\delta_{1\times 1}\left(\mathrm{cat}\left(d_{1},\varphi \left(d_{2}\right),\varphi \left(d_{3}\right),\varphi \left(d_{4}\right)\right)\right),$$

where $\delta_{k\times k}$ indicates a convolution with a kernel size of k×k, φ(·) denotes the interpolation operation, and $X\in R^{C\times \frac{H}{4}\times \frac{W}{4}}$ represents the merged feature of $d_{i=1}^{4}$ with a unified channel dimension C=64.

Then, the initial feature tensor *X* undergoes dual-domain decoupling. Specifically, *X* is decoupled into high-frequency and low-frequency features through wavelet transform frequency decoupling (WTFD). We employ the Haar wavelet and performed a two-level wavelet decomposition. The low-frequency components were used to capture the global variations of the image, while the high-frequency detail coefficients were employed to detect local changes. To minimize boundary artifacts, we used symmetric extension during the wavelet transform. During the spatial mapping process, *X* generates locally enhanced features via the SPP and globally enhanced features via the CA. This procedure can be formulated as:

(3) $$\left\{\begin{array}{c} F_{l},F_{h}=f_{w}\left(X\right),\\ F_{g}=f_{g}\left(X\right),\\ F_{m}=f_{m}\left(X\right), \end{array}\right.$$

where $f_{w}\left(\cdot \right)$, $f_{g}\left(\cdot \right)$, and $f_{m}\left(\cdot \right)$ represent the feature mapping operations for WTFD, CA, and SPP, respectively. $F_{l},F_{h},F_{g},F_{m}\in R^{C\times \frac{H}{4}\times \frac{W}{4}}$ represent the low-frequency features, high-frequency features, globally enhanced features, and locally enhanced features, respectively.

Spatial-domain and frequency-domain features are first categorized into two distinct groups. These features are then passed through the DAF and SFA modules, which address the semantic disparity between the two types for effective feature alignment. Finally, the aligned features, along with the output from the initial layer of the feature extraction stage, are fused. The combined features are subsequently processed by the segmentation head to generate the final CD map.

(4) $$\left.\left\{\begin{array}{c} F_{out}^{1}=f_{daf}\left(F_{m},F_{h}\right),\\ F_{out}^{2}=f_{sfa}\left(F_{g},F_{l}\right), \end{array}\right.\right.$$

(5) $$F_{out}=\delta_{1\times 1}(\mathrm{cat}\left(F_{out}^{1},F_{out}^{2},\varphi \left(d_{1}\right),X\right),$$

where $f_{daf}\left(F_{m},F_{h}\right)$ represents the DAF module, which aligns the local spatial features $F_{m}$ and the high frequency-domain features $F_{h}$; $f_{sfa}\left(F_{g},F_{l}\right)$ represents the SFA module used to fuse the global spatial features $F_{g}$ and the low frequency-domain features $F_{l}$; and $F_{out}^{1},F_{out}^{2}\in R^{C\times \frac{H}{4}\times \frac{W}{4}}$ are the outputs of the DAF and SFA modules. $F_{out}\in R^{C\times \frac{H}{4}\times \frac{W}{4}}$ denotes the final mixed feature.

### 3.2. MiT-Based Bi-Temporal Fusion Stage

Due to significant spatial misalignment, radiometric differences, and scale variations between bi-temporal images, simply stacking the two frames makes it challenging for the model to capture consistent change cues. To address this, we introduce MiT, a method that naturally enables cross-window interaction and convolutional local sensitivity. This hybrid attention mechanism effectively captures cross-scale dependencies while preserving the local structure. Additionally, the grouped mixing architecture facilitates the simultaneous optimization of both global and local feature representations. It effectively extracts rich semantic context while explicitly enhancing the model’s sensitivity to high-frequency spatial patterns, which are crucial for detecting changes. The process is as follows:

(6) $$\left[x_{i}^{t_{1}},x_{i}^{t_{2}}\right]=\mathrm{MiT}\left(\left[T_{1},T_{2}\right]\right).$$

Subsequently, we fuse the multi-layer features $x_{i}^{t_{1}},x_{i}^{t_{2}}$ into a spatiotemporally aligned joint representation, providing a unified base feature space for the subsequent frequency branches. This fusion effectively mitigates the unchanged noise arising from cross-temporal differences and accentuates the saliency of true changes. The process is as follows:

(7) $$d_{i}=\mathrm{cat}\left(x_{i}^{t_{1}},x_{i}^{t_{2}}\right),$$

where i=1,2,3,4 denotes the feature level and cat(·) represents the feature concatenation operation. The early fusion constructed by MiT provides a bi-temporal feature space with cross-scale consistency, laying the foundation for spatial-frequency decomposition.

### 3.3. Multi-Scale High-Frequency Interaction Module

Although the deep features extracted by the backbone network are rich in semantic information, their output represents a mix of various frequency components. The high-frequency details essential for change detection are often overshadowed by the dominant low-frequency semantic context. To effectively capture changes, it is necessary to explicitly isolate and enhance these high-frequency signals. As shown in Figure 2, we propose the multi-scale high-frequency interaction (MHI) module, which introduces an SPP unit to capture multi-scale local contexts, thereby ensuring sensitivity to subtle spatial perturbations. Meanwhile, the required high-frequency features are obtained using wavelet transform frequency decoupling (WTFD). Subsequently, a dual-domain alignment fusion (DAF) module is employed to model the long-range dependencies of high-frequency change patterns along the spatial dimension, enabling collaborative detection of continuous and structured change regions.

**Figure 3:**
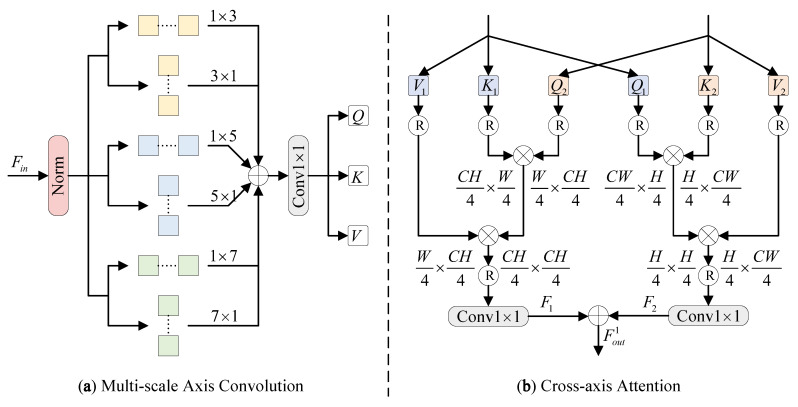
Architecture of the proposed DAF.

As shown in Figure 3a, to compensate for the limitation of axial attention in local information modeling, the input local-enhanced features and high-frequency features are first processed by the multi-scale axis convolution. This operation introduces strip-shaped convolution kernels with different kernel sizes multiple times along each axial attention path. Such a design effectively enhances the efficiency of the DAF module in encoding local information. The process is as follows:

(8) $$\begin{array}{c} Q,K,V=\delta_{1\times 1}\left(AC_{3}\left(LN\left(F_{in}\right)\right)+AC_{5}\left(LN\left(F_{in}\right)\right)+AC_{7}\left(LN\left(F_{in}\right)\right)\right), \end{array}$$

where $AC_{k}$ represents the axis convolution with kernel sizes of 1×k and k×1 and LN(·) represents layer normalization. $F_{in}$ represents the input features. Q,K,V are the matrices obtained after the multi-scale axis convolution operation.

As illustrated in Figure 3b, the $F_{m}$ and $F_{h}$ each produce three matrices, denoted as ($Q_{1}$, $K_{1}$, $V_{1}$) and ($Q_{2}$, $K_{2}$, $V_{2}$). To address the issues of insufficient positional bias learning and the limited long-range interactions in axial attention, a cross-axis attention mechanism is introduced. This mechanism computes attention by querying corresponding elements along with their respective key–value pairs. The computational steps involved in the cross-axis attention process are outlined as follows:

(9) $$\left.\left\{\begin{array}{c} F_{1}=\delta_{1\times 1}\left(Attn\left(Q_{2},K_{1},V_{1}\right)\right),\\ F_{2}=\delta_{1\times 1}\left(Attn\left(Q_{1},K_{2},V_{2}\right)\right), \end{array}\right.\right.$$

the attention formula is:

(10) $$Attn\left(Q,K,V\right)=Softmax\left(\frac{QK^{T}}{\sqrt{d}}\right)V,$$

where $\frac{1}{\sqrt{d}}$ denotes scaling factor.

(11) $$F_{out}^{1}=F_{1}+F_{2},$$

where $F_{1}$ and $F_{2}$ are the outputs of attention and $F_{out}^{1}$ is the output of the DAF module.

### 3.4. Position-Aware Low-Frequency Enhancement Module

Modern visual backbone networks, such as MiT, tend to weaken spatial location information when extracting high-level semantics through deep Transformer blocks. While their self-attention mechanisms effectively capture long-range dependencies, they rely less on absolute and relative position encoding. This leads to a blurred spatial layout for the output features, which hinders pixel-level accurate localization of change regions. To address this, we propose a position-aware low-frequency enhancement (PLE) module that explicitly compensates for and enhances the spatial and structural information of low-frequency features. As shown in Figure 2, CA decouples position information into 1D attention that is highly sensitive to the vertical direction, restoring the spatial layout expression of low-frequency features. Meanwhile, the required low-frequency features are obtained using wavelet transform frequency decoupling (WTFD). Then, the selective fusion attention (SFA) module provides a spatial-frequency selection mechanism to adaptively integrate spatial information and low-frequency structural information. Finally, a low-frequency change description that is most sensitive to large-area change regions is formed, significantly enhancing the representation and discrimination ability for low-frequency change signals.

**Figure 4:**
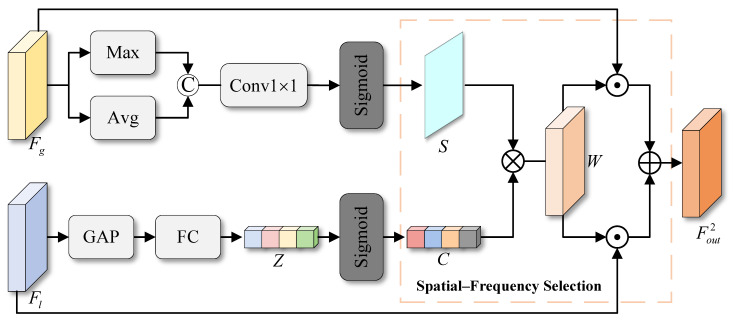
Architecture of the proposed SFA.

As shown in Figure 4, the spatial branch first applies channel-wise max pooling $P_{\max}\left(\cdot \right)$ and average pooling $P_{\mathrm{avg}}\left(\cdot \right)$ operations along the channel dimension to focus on the most salient and dominant features. To ensure information interaction between these two spatial descriptors, the pooled features are initially concatenated. Subsequently, a simple 1×1 convolutional layer is employed to map these two pooled features into two spatial attention maps. The above process can be expressed as follows:

(12) $$\bar{S}=\delta_{1\times 1}(\mathrm{cat}\left(P_{\max}\left(F_{g}\right),P_{\mathrm{avg}}\left(F_{g}\right)\right),$$

where the spatial attention feature maps $\bar{S}\in R^{2\times \frac{H}{4}\times \frac{W}{4}}$. Next, the sigmoid activation function σ(·) is applied to obtain a spatial selection mask *S*:

(13) $$S=\sigma (\bar{S}).$$

In frequency domain analysis, signals are decomposed into different frequency components. Low-frequency features typically represent smooth, structured regions in an image, capturing the overall contours and background. The variations in these regions are small and often repeat across multiple local areas. As a result, low-frequency features tend to have redundant responses in different parts of the image. This redundancy does not increase the effective information in the image. Instead, it can cause overfitting, increase computational burden, and reduce the model’s performance.

The frequency-domain selection mechanism addresses this by evaluating the importance of each frequency component. It suppresses redundant low-frequency information and focuses on more discriminative high-frequency features, such as edges and fine details. These high-frequency features contain more local variations and valuable information, helping the model make more accurate decisions. Specifically, global average pooling $P_{\mathrm{GAP}}\left(\cdot \right)$ is first applied to compress the feature $F_{l}$, producing a frequency-aware feature descriptor. Subsequently, a fully connected layer $F_{\mathrm{FC}}\left(\cdot \right)$ generates a more compact frequency-domain attention feature $Z\in R^{C\times 1}$. This process can be formulated as:

(14) $$Z=F_{\mathrm{FC}}\left(P_{\mathrm{GAP}}\left(F_{l}\right)\right).$$

Then, we apply a sigmoid operation to the low-frequency attention feature *Z* to generate frequency-domain attention weights. These weights adaptively modulate the low-frequency channels, producing a frequency selection mask $C\in R^{C\times 1}$. This process suppresses redundant low-frequency responses while emphasizing more discriminative low-frequency structural components. The process is outlined as follows:

(15) C=σ(Z).

Subsequently, the corresponding spatial–frequency selection masks are multiplied to obtain the spatial–frequency selection weight:

(16) W=S×C.

Finally, the global feature $F_{g}$ and low-frequency feature $F_{l}$ are weighted by the spatial-frequency selection weight *w*. The final output is generated after summation:

(17) $$F_{out}^{2}=F_{g}\times W+F_{l}\times W.$$

### 3.5. Loss Function

The loss function L is a combination of the dice coefficient (Dice) loss $L_{\mathrm{Dice}}$ [66] and the binary cross-entropy (BCE) loss $L_{\mathrm{BCE}}$ [67]. The formula can be written as follows:

(18) $$L_{\mathrm{BCE}}\left(p,g\right)=g\log\left(p\right)+\left(1-g\right)\log\left(1-p\right),$$

(19) $$L_{\mathrm{Dice}}\left(p,g\right)=1-\frac{2gp}{∥g∥+∥p∥},$$

(20) $$L\left(p,g\right)=L_{\mathrm{BCE}}\left(p,g\right)+L_{\mathrm{Dice}}\left(p,g\right),$$

where *g* and *p* represent the ground-truth labels and the corresponding predicted change maps, respectively. The pixel values in *g* are either 0 or 1, while the pixel values in *p* range from 0 to 1, indicating the probability of being predicted as changed.

## 4. Experiment

### 4.1. Datasets

#### 4.1.1. WHU-CD Dataset

The WHU-CD [68] dataset is a building CD dataset consisting of a pair of high-resolution aerial images, each with a resolution of 0.2 m and pixel dimensions of 32,507 × 15,354. The dataset covers a city in New Zealand and focuses on detecting changes in buildings. The complex building structures make the precise detection of building boundaries challenging. We divided the images into non-overlapping patches of size 256 × 256 and split them into 5947 training samples, 743 validation samples, and 744 testing samples.

#### 4.1.2. LEVIR-CD Dataset

LEVIR-CD [69] provides 637 paired high-resolution images (1024 × 1024 pixels, 0.5 m resolution) collected from 20 distinct urban areas in Texas, USA. It is widely used for detecting notable changes in land use, particularly related to building activity. The dataset includes a broad spectrum of building categories, from villas and high-rise apartments to smaller structures and large storage facilities. The LEVIR-CD dataset also contains a significant amount of seasonal and lighting changes, which make CD more challenging. We divided the images into non-overlapping patches of size 256 × 256 and split them into 7120 training samples, 1024 validation samples, and 2048 testing samples.

#### 4.1.3. SYSU-CD Dataset

The SYSU-CD [70] dataset includes land cover change data from Hong Kong spanning from 2007 to 2014. It comprises 20,000 pairs of VHR RS images. The SYSU-CD dataset covers a variety of complex change types such as buildings, urban roads, ports, and ships, as well as mountainous vegetation, with significant interference from pseudo-changes. We divided the images into non-overlapping patches of size 256 × 256 and split them into 12,000 training, 4000 validation, and 4000 testing samples.

### 4.2. Implementation Details

In the experiments, the network was trained with the PyTorch 1.7.1 framework on two NVIDIA RTX 2080Ti GPUs. The AdamW optimizer was used with the following settings: a batch size of 16, an initial learning rate of 0.0001, momentum of 0.99, epochs of 200, and weight decay of 0.01. A linear learning rate decay strategy was implemented. To ensure the reproducibility of the experiments, we fixed the random seed to 42 in all experiments and will set this in the code. Data augmentation was applied to all experimental data during the training phase to improve robustness and generalization. The techniques included flipping, cropping, and Gaussian blurring.

### 4.3. Evaluation Metrics

To evaluate the performance of CD methods quantitatively, we use five widely adopted metrics: precision (Pre), recall (Rec), F1-score (F1), intersection over union (IoU), and overall accuracy (OA). The definitions of these metrics are provided below:

(21) Pre=TP/(TP+FP),

(22) Rec=TP/(TP+FN),

(23) F1=2×Rec×Pre/(Rec+Pre),

(24) IoU=TP/(TP+FP+FN),

(25) OA=(TP+TN)/(TP+TN+FP+FN),

where TP, TN, FP, and FN denote the number of true positives, true negatives, false positives, and false negatives, respectively.

### 4.4. Compared Methods

To validate the effectiveness of SDA-Encoding in RSCD tasks, this section selects eight state-of-the-art models as competitors. These models include three methods based on FCN: FC-EF [47], FC-Siam-Diff [47], and FC-Siam-Conc [47]; four models based on attention mechanisms: DMINet [48], SEIFNet [49], SChanger [71], and CASP [72]; and three models based on Transformers: BIT [38], ChangFormer [52], and STADE-CDNet [53]. Each method was retrained under identical experimental conditions, strictly adhering to the official implementations provided by the respective authors.

### 4.5. Results and Analysis

#### 4.5.1. Results on the WHU-CD Dataset

Table 1 presents the results of generalization experiments conducted on the WHU-CD dataset. Among all compared methods, SDA-Encoding achieves the best overall performance with an IoU of 89.87% and F1-score of 94.67%, outperforming other state-of-the-art approaches. Specifically, SDA-Encoding demonstrates a balanced trade-off between a precision of 96.69% and a recall of 92.73%, indicating its superior capability in accurately identifying change regions while maintaining low false detection rates.

**Figure 5:**
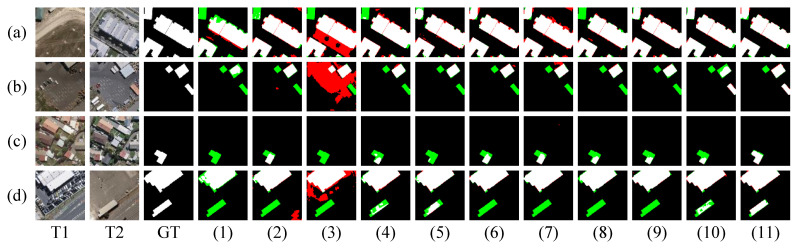
Visualization results of different methods on the WHU-CD test set. (**a**–**d**) denote prediction results of all the compared methods for different samples, respectively. (1–11) represent FC-EF, FC-Siam-Diff, FC-Siam-Conc, BIT, ChangeFormer, DMINet, SEIFNet, STADE-CDNet, SChanger, CASP, and SDA-Encoding. White denotes TP, black denotes TN, red denotes FP, and green denotes FN.

As shown in Figure 5a, although significant changes are visually apparent, most existing methods fail to detect them accurately. This is primarily because, from a semantic perspective, these methods do not identify such areas as building changes. While some approaches can detect the changes, they still experience considerable false positives and missed detections along object boundaries. In contrast, our method, which incorporates frequency-domain learning, effectively captures the structural and edge features of the changed objects, resulting in more precise detection. As shown in Figure 5b, although the FC-Siam-Conc method detected the change in the upper-left corner, it was accompanied by a significant number of false positives. In contrast, other methods failed to identify this change. This suggests that our method struggles to detect changes in buildings with high ambiguity, highlighting a key limitation of our approach. As shown in Figure 5c,d, when the background is complex or when the foreground and background share similar colors, only our method successfully detects the changed objects. This demonstrates that the joint learning of low-frequency structural features and high-frequency edge features enhances the model’s robustness in complex change detection scenarios.

#### 4.5.2. Results on the LEVIR-CD Dataset

Table 2 reports the generalization results on the LEVIR-CD dataset. As shown, the proposed SDA-Encoding achieves the best results across all evaluation metrics. Compared with previous methods such as ChangeFormer and DMINet, SDA-Encoding significantly improves IoU and F1 scores, indicating its superior capability in accurately capturing both semantic consistency and fine-grained structural changes.

**Figure 6:**
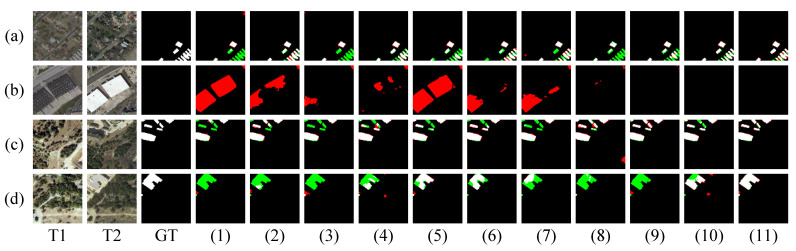
Visualization results of different methods on the LEVIR-CD test set. (**a**–**d**) denote prediction results of all the compared methods for different samples, respectively. (1–11) represent FC-EF, FC-Siam-Diff, FC-Siam-Conc, BIT, ChangeFormer, DMINet, SEIFNet, STADE-CDNet, SChanger, CASP, and SDA-Encoding. White denotes TP, black denotes TN, red denotes FP, and green denotes FN.

As shown in Figure 6a,c,d, when dealing with blurred foreground–background boundaries and small-scale change targets, only our method can fully and accurately detect the change regions. This suggests that our approach not only preserves sufficient spatial information but also integrates frequency-domain features, enhancing the detection of fine-grained changes. Furthermore, as shown in Figure 6b, when pseudo-changes such as color variations occur, our method effectively suppresses disturbances caused by color and seasonal changes in the spatial domain by leveraging frequency-domain structural information. This enables the model to capture more precise semantic representations of the changed objects.

#### 4.5.3. Results on the SYSU-CD Dataset

Table 3 summarizes the generalization results on the SYSU-CD dataset. As shown, SDA-Encoding achieves the best overall performance among all compared methods, reaching an IoU of 71.57% and an F1-score of 83.43%. Compared to other strong baselines such as DMINet, SChanger, and CASP, SDA-Encoding achieves notable improvements in both precision (which was 87.46%) and IoU, demonstrating its robustness and superior generalization capability when transferring to a new domain with complex urban and rural scene variations. Although STADE-CDNet exhibits a slightly higher recall of 83.11%, its overall accuracy and precision are significantly lower, indicating a tendency toward over-detection. These results confirm that the multi-scale dual-attention mechanism in SDA-Encoding effectively enhances the model’s ability to distinguish subtle and diverse change patterns under challenging domain shifts.

**Figure 7:**
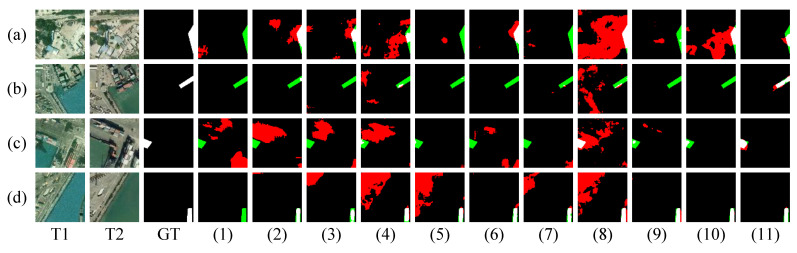
Visualization results of different methods on the SYSU-CD test set. (**a**–**d**) denote prediction results of all the compared methods for different samples, respectively. (1–11) represent FC-EF, FC-Siam-Diff, FC-Siam-Conc, BIT, ChangeFormer, DMINet, SEIFNet, STADE-CDNet, SChanger, CASP, and SDA-Encoding. White denotes TP, black denotes TN, red denotes FP, and green denotes FN.

As shown in Figure 7, the two previously discussed datasets are limited to binary change detection (CD) tasks, whereas the SYSU-CD dataset involves multi-class change scenarios and introduces additional irrelevant background variations. Experimental results reveal that existing methods tend to generate a large number of irrelevant or non-target change predictions. In contrast, our proposed SDA-Encoding significantly outperforms these methods by effectively suppressing such spurious changes. This improvement is largely attributed to the integration of spatial and frequency-domain features. However, it is important to note that our method encounters challenges when handling datasets with multiple types of changes. Despite the changes being clearly visible, the method struggles to accurately segment the changed objects. This suggests that our approach has not yet fully captured the semantic information related to different change types, leading to confusion and limitations in performance.

### 4.6. Ablation Studies

As shown in Table 4, ablation studies were conducted on the LEVIR-CD dataset to validate the impact of different components and configurations of SDA-Encoding. These experiments aim to assess the significance of individual components by systematically removing them and observing the resulting performance changes.

Table 4 presents the results of the ablation study conducted on the LEVIR-CD dataset, evaluating the contribution of each proposed component, including the MHI, PLE, DAF, and SFA modules. When two modules are combined, the performance improves further, highlighting their complementary roles in enhancing feature representation and change localization. Ultimately, the integration of all four modules yields the best results, with an IoU of 85.29% and an F1 score of 92.06%. This demonstrates that the MHI, PLE, DAF, and SFA modules work together to boost both global semantic understanding and fine-grained structural perception, resulting in more accurate and robust change detection.

Table 5 presents the experimental results of various backbone networks used as feature extractors on the LEVIR-CD dataset. The performance varies across different backbones. Notably, ViT [36], Swin-T [37], MobileViT [73], and FastViT [74] show slightly lower performance. This suggests that MiT better captures multi-scale features and global contextual information, leading to more accurate identification and delineation of change regions.

**Figure 8:**
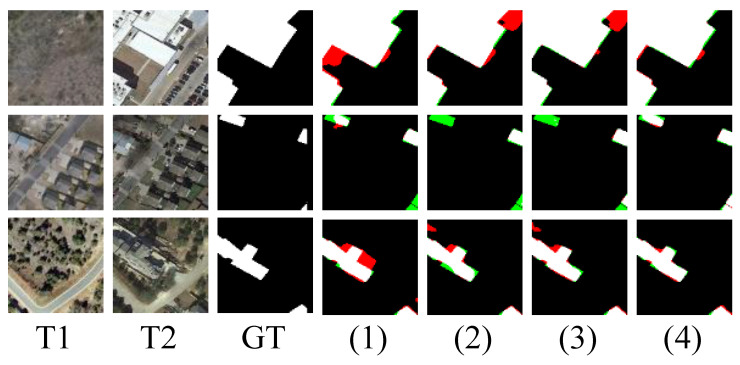
The figure shows the locally enlarged segmentation results after removing different components from the SDA-Encoding module. (1) represents SDA-Encoding without the PLE module. (2) represents SDA-Encoding without the MHI module. (3) represents SDA-Encoding without frequency information. (4) represents SDA-Encoding. White denotes TP, black denotes TN, red denotes FP, and green denotes FN.

Figure 8 illustrates the effects of adding each individual component to the baseline model. Overall, the results demonstrate that each component proposed in our study has a positive impact on the model’s performance.

**Figure 9:**
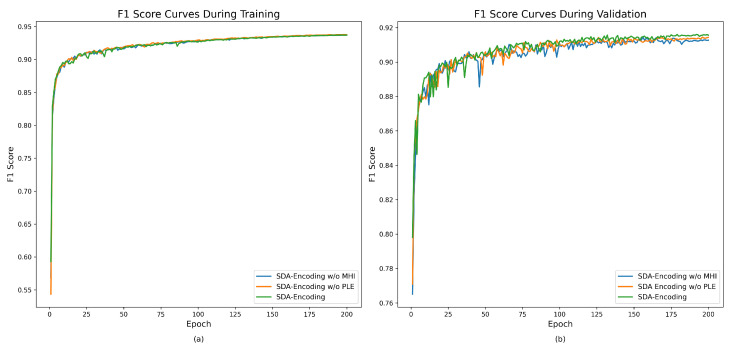
F1-score curves of SDA-Encoding during the training epochs. (**a**) Training F1-score on the LEVIR-CD dataset. (**b**) Validation F1-score on the LEVIR-CD dataset.

Figure 9 illustrates the changes in F1 scores over training epochs for three different SDA-Encoding methods during both training and validation. It shows that the F1 scores for all methods rise rapidly in the early training stages and then gradually converge. Overall, SDA-Encoding and SDA-Encoding w/o PLE exhibit similar, stable performance, while SDA-Encoding w/o MHI also maintains strong and steady performance in both training and validation. This suggests that the MHI and PLE modules both influence model performance, but all methods steadily improve as training progresses.

### 4.7. Comparison of Complexity

Table 6 compares the model complexity across different methods. Our proposed SDA-Encoding model has 45.57 M parameters, 44.80 G FLOPs, and 90.51 samples per second.

Compared to lightweight approaches, our model is more complex. This is mainly due to the dual-branch encoder, which is designed to capture multi-scale spatiotemporal variations. Despite its higher complexity, our model achieves high accuracy in change detection tasks. It performs especially well in detecting subtle texture differences and complex lighting changes. Remote sensing image change detection often requires capturing fine local textures and long-range edge information. More complex network architectures can improve the recognition accuracy of these details. Although our model has more parameters than other methods, its superior accuracy makes it highly valuable for high-precision remote sensing change detection. This is particularly true for detecting changes in complex images or extreme conditions. However, in real-world applications, such as real-time remote sensing monitoring with drones or satellites, computational resources may be limited. Reducing computational cost while maintaining high accuracy is an ongoing challenge.

Therefore, our follow-up work will focus on optimizing the model architecture. This includes exploring more efficient Transformer structures or convolutional operators to replace the existing MiT encoder. Additionally, we aim to compress the model size via pruning and knowledge distillation without significantly sacrificing performance. We will also investigate sparse activation strategies for specific tasks, enabling high-frequency feature interactions only in key regions.

## 5. Conclusions

In this paper, we proposed a spatial-frequency decoupling alignment encoding (SDA-Encoding) for change detection in bi-temporal remote sensing imagery. The proposed framework effectively integrates spatial-domain and frequency-domain representations to capture both fine-grained texture variations and large-scale structural changes. Specifically, a Transformer-based bi-temporal fusion stage constructs cross-scale consistent spatial features, serving as the foundation for spatial–frequency decoupling. The multi-scale high-frequency interaction (MHI) module enhances local texture sensitivity through spatial pyramid pooling (SPP) and employs a dual-domain alignment fusion (DAF) module to model spatial and cross-scale dependencies. Meanwhile, the position-aware low-frequency enhancement (PLE) module incorporates coordinate attention to explicitly encode positional information and utilizes a selective fusion attention (SFA) module to strengthen global structural awareness. Finally, a segmentation head aggregates multi-scale features to generate precise change detection maps. Extensive experiments on multiple benchmark datasets demonstrate that SDA-Encoding achieves superior performance compared with existing state-of-the-art methods. The results confirm the effectiveness of the proposed spatial–frequency decoupling strategy and the dual-branch architecture, which enhance both detail preservation and structural consistency.

In future work, we plan to explore weakly supervised learning strategies to reduce the dependence on fine-grained annotations and further investigate the potential of frequency-domain features in RSCD. Specifically, we aim to further investigate the potential of frequency-domain features in RSCD. In weakly supervised settings, pseudo-labels are often used for training. Since frequency-domain features are better at distinguishing real structural changes from low-frequency noise (such as lighting and shadows), pseudo-labels generated from these features are likely to be cleaner and more reliable than those based solely on spatial-domain features. This could improve the performance limits of weakly supervised learning. Additionally, we plan to streamline the network architecture to maintain performance while enhancing the model’s practicality and deployment efficiency.

## Figures and Tables

**Table 1:** Generalization experiments conducted on the WHU-CD dataset. The best results are indicated in bold.

Methods	OA (%)	IoU (%)	F1 (%)	Pre (%)	Rec (%)
FC-EF	98.19	58.30	73.66	87.25	63.73
FC-Siam-Diff	98.25	67.34	80.48	72.27	90.80
FC-Siam-Conc	95.91	45.37	62.42	49.08	85.72
BIT	99.12	79.44	88.54	91.46	85.81
ChangeFormer	99.11	79.16	88.37	92.21	84.83
DMINet	98.52	71.19	83.17	75.79	92.14
SEIFNet	98.80	75.12	85.79	81.12	91.04
STADE-CDNet	99.20	80.77	89.36	94.06	85.11
SChanger	99.16	80.86	89.42	89.68	89.16
CASP	99.54	88.71	94.02	**96.94**	91.27
SDA-Encoding	**99.59**	**89.87**	**94.67**	96.69	**92.73**

**Table 2:** Generalization experiments conducted on the LEVIR-CD dataset. The best results are indicated in bold.

Methods	OA (%)	IoU (%)	F1 (%)	Pre (%)	Rec (%)
FC-EF	98.38	72.19	83.85	85.10	82.63
FC-Siam-Diff	98.98	81.38	89.74	92.09	87.50
FC-Siam-Conc	98.62	75.72	86.18	87.83	84.59
BIT	98.99	81.39	89.74	92.66	87.01
ChangeFormer	99.00	81.60	89.87	91.88	87.95
DMINet	99.03	82.27	90.27	92.42	88.22
SEIFNet	98.91	80.16	88.99	91.79	86.36
STADE-CDNet	98.91	80.44	89.16	90.65	87.72
SChanger	99.04	82.25	90.26	92.99	87.69
CASP	99.10	83.47	90.99	92.50	89.53
SDA-Encoding	**99.20**	**85.29**	**92.06**	**93.34**	**90.82**

**Table 3:** Generalization experiments conducted on the SYSU-CD dataset. The best results are indicated in bold.

Methods	OA (%)	IoU (%)	F1 (%)	Pre (%)	Rec (%)
FC-EF	89.57	61.81	76.40	81.93	71.57
FC-Siam-Diff	91.37	67.52	80.61	85.70	76.09
FC-Siam-Conc	90.06	62.99	77.29	83.74	71.77
BIT	89.31	62.41	76.85	78.49	75.28
ChangeFormer	90.51	65.18	78.92	82.89	75.31
DMINet	91.78	69.11	81.73	85.85	78.00
SEIFNet	91.59	69.32	81.88	83.22	80.59
STADE-CDNet	87.11	60.33	75.26	68.76	**83.11**
SChanger	91.62	69.24	81.83	83.73	80.01
CASP	91.67	68.79	81.51	85.51	77.87
SDA-Encoding	**92.53**	**71.57**	**83.43**	**87.46**	79.75

**Table 4:** Ablation study of different modules. The best results are indicated in bold.

Baseline	MHI	PLE	DAF	SFA	LEVIR-CD				
					OA (%)	IoU (%)	F1 (%)	Pre (%)	Rec (%)
✓	×	×	×	×	99.00	81.60	89.87	91.88	87.95
✓	×	✓	×	✓	99.15	84.42	91.55	92.17	**90.95**
✓	✓	×	✓	×	99.14	84.31	91.49	92.40	90.60
✓	✓	✓	×	×	99.06	83.06	90.75	91.34	90.16
✓	×	×	✓	✓	99.14	84.26	91.46	92.12	90.81
✓	✓	✓	✓	✓	**99.20**	**85.29**	**92.06**	**93.34**	90.82

**Table 5:** The experimental results of different backbones on the LEVIR-CD dataset. The best results are indicated in bold.

Method	LEVIR-CD				
	OA (%)	IoU (%)	F1 (%)	Pre (%)	Rec (%)
Vit	99.08	83.35	90.91	91.28	90.55
Swin-T	99.14	84.25	91.45	92.62	90.32
MobileVit	99.14	84.45	91.57	92.74	90.43
FastVit	99.08	83.47	90.99	90.97	**91.01**
MiT-b0	99.15	84.52	91.61	92.47	90.76
MiT-b3	**99.20**	**85.29**	**92.06**	**93.34**	90.82

**Table 6:** Complexity comparison of different methods.

Methods	Para. (M)	FLOPs (G)	Throughput (Sample/s)
FC-EF	1.35	3.59	721.87
FC-Siam-conc	1.55	5.34	566.48
FC-Siam-diff	1.35	4.74	577.64
BIT	12.40	10.87	237.35
Changeformer	41.04	45.97	148.40
DMINet	6.24	14.55	215.29
SEIFNet	27.91	8.37	292.40
STADE-CDNet	11.90	10.25	63.68
SChanger	2.37	17.91	87.36
CASP	14.55	9.19	91.47
SDA-Encoding (Ours)	45.57	44.80	90.51

## Data Availability

The data presented in this study are available upon request from the corresponding author.
